# MNSFβ Regulates TNFα Production by Interacting with RC3H1 in Human Macrophages, and Dysfunction of MNSFβ in Decidual Macrophages Is Associated With Recurrent Pregnancy Loss

**DOI:** 10.3389/fimmu.2021.691908

**Published:** 2021-09-13

**Authors:** Xing-Xing Zhen, Long Yang, Yan Gu, Qian Yang, Wen-Wen Gu, Ya-Ping He, Yan-Ling Wang, Jian Wang

**Affiliations:** ^1^National Health Commission (NHC) of the People’s Republic of China Key Lab of Reproduction Regulation (Shanghai Institute for Biomedical and Pharmaceutical Technologies), School of Pharmacy, Fudan University, Shanghai, China; ^2^State Key Laboratory of Stem Cell and Reproductive Biology, Institute of Zoology, Chinese Academy of Sciences, Beijing, China; ^3^Department of Gynecology and Obstetrics, The Second Hospital of Tianjin Medical University, Tianjin, China; ^4^Beijing Institute of Stem Cell and Regenerative Medicine, Beijing, China; ^5^Savaid Medical School, University of Chinese Academy of Sciences, Beijing, China

**Keywords:** decidual macrophages, RC3H1, TNFα, MNSFβ, recurrent pregnancy loss

## Abstract

Decidual macrophages (dMϕ) are the second largest population of leukocytes at the maternal–fetal interface and play critical roles in maintaining pregnancy. Our previous studies demonstrated the active involvement of monoclonal nonspecific suppressor factor-β (MNSFβ) in embryonic implantation and pregnancy success. MNSFβ is a ubiquitously expressed ubiquitin-like protein that also exhibits immune regulatory potential, but its function in human dMϕ remains unknown. Here, we observed that the proportion of CD11c^high^ (CD11cHI) dMϕ was significantly increased in dMϕ derived from patients with recurrent pregnancy loss (RPL dMϕ) compared to those derived from normal pregnant women (Control dMϕ). The production of MNSFβ and TNFα by RPL dMϕ was also significantly increased compared to that by Control dMϕ. Conditioned medium from RPL dMϕ exerted an inhibitory effect on the invasiveness of human trophoblastic HTR8/SVneo cells, and this effect could be partially reversed by a neutralizing antibody against TNFα. Bioinformatics analysis indicated a potential interaction between MNSFβ and RC3H1, a suppressor of TNFα transcription. Immunoprecipitation experiments with human Mϕ differentiated from the human monocyte cell line Thp1 (Thp1-derived Mϕ) proved the binding of MNSFβ to RC3H1. Specific knockdown of MNSFβ in Thp1-derived Mϕ led to a marked decrease in TNFα production, which could be reversed by inhibiting RC3H1 expression. Interestingly, a significant decrease in the protein level of RC3H1 was observed in RPL dMϕ. Together, our findings indicate that aberrantly increased MNSFβ expression in dMϕ may promote TNFα production *via* its interaction with RC3H1, and these phenomena could result in the disruption of the immune balance at the maternal–fetal interface and thus pregnancy loss.

## Introduction

The establishment and maintenance of maternal–fetal tolerance, mediated by fetal and maternal cells, including extravillous trophoblasts (EVTs), decidual stromal cells (DSCs), and decidual immune cells (DICs), are crucial for successful human pregnancy (1). The most abundant cell types among DICs are decidual NK cells (dNK, 50%–70%), decidual macrophages (dMϕ, 20%–30%), and T cells (10%–15%) (2). Decidual macrophages have been thought to play critical roles at the maternal–fetal interface, including roles in vascular remodeling (3), cell debris clearance (4), and parturition (5); however, the exact roles of dMϕ remain largely unknown.

MNSFβ (monoclonal nonspecific suppressor factor-β), also known as Fau (Finkel-Biskis-Reilly murine sarcoma virus-associated ubiquitously expressed gene), is a 133-aa protein containing a ubiquitin-like (Ubi-L/FUBI) domain and a ribosomal protein S30 domain, and the homology between the murine and human MNSFβ proteins is greater than 97.8% (6). MNSFβ was originally identified as an inhibitor of the T-cell-mediated immune response (7) because it inhibits not only the proliferation of T and B cells but also the secretion of interleukin-4 (IL-4) from type 2 helper T cells and bone marrow-derived mast cells (8, 9). Furthermore, it has been reported that MNSFβ promotes the apoptosis (10) but inhibits the phagocytosis (11) and TNFα production (12) of murine macrophages.

MNSFβ was first shown to be involved in embryo implantation due to its differential expression between the implantation sites and interimplantation sites of endometrial tissues in mice (13). Subsequently, it was revealed by our previous studies that deficiency in MNSFβ could lead to embryo implantation failure in mice (14, 15), and the MNSFβ expression levels in both the decidual and villus tissues of RPL patients were significantly decreased (16, 17). In particular, MNSFβ exerted stimulatory effects on the proliferation and migration of human EVTs, suggesting that insufficient MNSFβ expression at the maternal–fetal interface might lead to early pregnancy loss by interfering with the invasion of EVTs (17).

Given that MNSFβ is widely expressed in various tissues and cells (6), and it is involved in regulation of embryo implantation, as well as the immune response of macrophages, we supposed that MNSFβ might contribute to the immune balance at the maternal–fetal interface by regulating activities of dMϕ, and malfunction of MNSFβ in dMϕ might be associated with the early pregnancy failure. Thus, this study was carried out to investigate the alteration of MNSFβ expression in dMϕ of RPL patients, and the effect and its underlying molecular pathway of abnormal MNSFβ expression on activities of dMϕ by using bioinformatic analysis and a human macrophage model differentiated from immortalized human monocyte cells.

## Materials and Methods

### Human Decidual Tissue Collection

Human decidual tissues from 24 RPL patients (RPL, 6–10 weeks of gestation) and 25 normal pregnant women in the first trimester (Control, 6–9 weeks of gestation) were collected at the Department of Gynecology and Obstetrics, the Second Hospital of Tianjin Medical University (Tianjin, China). These collected decidual tissues were immediately washed several times with sterile, glucose-free PBS solution until there were no obvious blood clots in the decidual tissues. Then, the tissues were immersed in ice-cold RPMI-160 medium. Cells were harvested from the tissues or the tissues were fixed within 3 h. Current pregnancy losses of the RPL patients were objectively confirmed by transvaginal ultrasound examination. Patients with classical risk factors, including abnormal parental karyotypes, uterine anatomical abnormalities, infectious diseases, luteal phase defects, diabetes mellitus, thyroid dysfunction, and hyperprolactinemia, were excluded from this study. In parallel, Control women who had no history of miscarriage and were undergoing legal, voluntary terminations of early pregnancy were enrolled and evaluated for classical risk factors for early pregnancy loss. The sample collection for this study was approved by the Medical Ethics Committees of The Second Hospital of Tianjin Medical University (KY2017K002) and Shanghai Institute for Biomedical and Pharmaceutical Technologies (former Shanghai Institute of Planned Parenthood Research) (Ref # 2018-05). All the samples were collected after informed consent was obtained. No significant differences in the average age or gestational week at sampling were observed between the RPL patients and Control women (**Supplementary Table S1**).

### Isolation of Human Decidual Macrophages (dMϕ)

DMϕ were isolated as previously described (18, 19) with modifications. Briefly, tissues were washed and crushed into small pieces by a tissue crusher (Gentle MACS Dissociator, Miltenyi Biotec, Germany). Pieces of decidual tissues were digested in 3 mg/ml collagenase Type IV (Gibco, USA), 100 IU/ml DNase I (Sigma-Aldrich, USA), 100 IU/ml penicillin, and 100 IU/ml streptomycin (Gibco) at 37°C for 30 min. Subsequently, the decidual cells that were released were filtered through 100, 200, and 400 mesh sieves (Corning, USA). To exclude any remaining red blood cells, the filtered cells were incubated with red blood cell lysis buffer (BD Biosciences, USA). DMϕ were obtained with CD14+ antibodies conjugated to magnetic beads (Miltenyi Biotec). The purity of the dMϕ (CD45^+^ CD14^+^), which was detected by flow cytometry, was approximately 90% (**Supplementary Figure S1B**).

### Cell Culture

Primary dMϕ was cultured at a final concentration of 1 × 10^6^ cells/ml in RPMI 1640 medium supplemented with 10% fetal bovine serum (FBS), 100 U/ml penicillin, and 100 µg/ml streptomycin (Gibco) under standard culture conditions (37°C in a 5% humidified CO_2_ incubator). The immortalized human first-trimester extravillous trophoblast cell line, HTR8/SVneo, characterized by the abilities of growth and invasion (20), were kindly provided by Prof. Hongmei Wang, Institute of Zoology, Chinese Academy of Sciences, China, and cultured in RPMI 1640 medium, supplemented with 10% FBS, 100 U/ml penicillin, and 100 µg/ml streptomycin (Gibco) under standard culture conditions (37°C in a 5% humidified CO_2_ incubator). The immortalized human monocyte cell line, Thp1, characterized by the absence of immunoglobulins, and the ability to restore T-lymphocyte response to ConA (21), was purchased from the American Type Culture Collection (ATCC, Manassas, USA). Thp1 cells were cultured routinely in RPMI 1640 medium plus 10% FBS and 0.1% β-mercaptoethanol with antibiotics (Gibco) under standard culture conditions (37°C in a 5% humidified CO_2_ incubator) and treated with 200 ng/ml phorbol myristate acetate (PMA) for 24 h to induce the differentiation of Thp1 cells into macrophages. Thp1-derived Mϕ cells differentiate normally and expressed high level of the macrophage markers CD11b and SPI1 (**Supplementary Figure S2**).

### Immunofluorescence Staining and Confocal Microscopy

Fresh decidual tissues collected from physically normal pregnant women in the early stage of pregnancy (8W) were embedded in Tissue-Tek O.C.T. compound (Sakura Finetek, USA), and frozen sections were generated with a thickness of 8 µm. The frozen sections were fixed in 4% paraformaldehyde (Sigma-Aldrich) for 24 h at 4°C and treated with 0.1% Triton. Then, the frozen sections were incubated with antibodies against human MNSFβ (0.5 mg/ml, prepared by our lab), CK7 (0.5 mg/ml, ZSGB-BIO, China), CD31 (1 μg/ml, Abcam, USA), or CD14 (1 μg/ml, Abcam). Then, the sections were incubated with FITC-conjugated or TRITC-conjugated secondary antibodies (ZSGB-BIO, China), and the cell nuclei of the sections were stained with 4,6-diamidino-2-phenylindole (DAPI; Sigma Aldrich). The results were recorded using a laser confocal microscope (Leica, Germany) and processed with ZEN 2012 software (Zeiss).

### Real-Time Reverse Transcription Polymerase Chain Reaction Analysis

Total cellular RNA was extracted using TRIzol (Invitrogen) according to the manufacturer’s instructions. Reverse transcription was performed with 0.2 μg of total RNA using SuperScript II Reverse Transcriptase (Invitrogen) and an oligo-dT primer (Invitrogen). Detailed information about the sequences of the primers is listed in **Supplementary Table S2**. Real-time PCR was performed by using the SYBR II kit (TaKaRa, China) according to the manufacturer’s instructions on a Light Cycler 480 real-time PCR System (Roche, USA). The relative mRNA expression levels were determined by the 2^−ΔΔCt^ method and normalized to the expression levels of Gapdh.

### Western Blotting Analysis

Total cellular proteins were extracted from dMϕ or decidual cells using TRIzol (Invitrogen, USA) or SDS lysis buffer (2% SDS, 50 mM Tris-HCI, pH 7.6, 2 mM EDTA, and 10% glycerol). The protein concentrations were quantified by using the BCA Protein Assay Kit (Thermo Pierce, USA). Then, 20 μg of protein was subjected to 10% SDS-PAGE and electrophoretically transferred to a PVDF membrane (Thermo Pierce). After blocking with 5% BSA, the membrane was incubated with different primary antibodies (**Supplementary Table S3**) overnight at 4°C. Then, the membrane was washed and incubated with the corresponding horseradish peroxidase-conjugated secondary antibodies for 1 h at room temperature. The results were captured with a Gene Gnome Imaging System (Syngene, UK). The relative densities of the target proteins were determined by normalization to the density of beta-actin (β-actin) in the same blot, and the results were analyzed with ImageJ software.

### Flow Cytometry

Cells were incubated with Human TruStainFcX (BioLegend, USA) for 30 min to block the Fc receptors and subsequently stained with CD45-Pacific Blue, CD14-APC, and CD11c-FITC (BioLegend) for 30 min at 4°C. After washing with PBS three times, the cells were incubated with Cytofix/Cytoperm (BD Biosciences) for 20 min at 4°C. After washing with wash buffer (BD Biosciences) three times, the cells were incubated with specific antibodies against MNSFβ for 30 min at 4°C. Then, the cells were incubated with Cyanine Cy™5-conjugated secondary antibodies (Jackson, USA) for 30 min at 4°C. Approximately 100,000 cells were detected using FACS (BD, USA), and the data were analyzed with FlowJo V10.2.

### Small Interfering RNA Transfection in Macrophages

SiRNA against MNSFβ (siMNSFβ, sense 5’-3’: CCAAACAGGAGAAGAAGAATT, antisense 3’-5’: UUCUUCUUCUCCUGUUUGGTT) (Genepharma, China) or siRNA against RC3H1 (sense 5’-3’: CGUGUUGUAAACUCUCAGUAU, antisense 3’-5’: AUACUGAGAGUUUACAACACG) (Genepharma, China) was used to knock down the expression level of MNSFβ or RC3H1 in macrophages, respectively, and the scrambled siRNA (NC, sense 5’-3’: UUCUCCGAACGUGUCACGUTT, antisense 3’-5’: ACGUGACACGUUCGGAGAATT) (Genepharma, China) was used as the negative control. Thp1 cells were treated with 200 ng/ml PMA for 24 h to generate macrophages, and the Thp1-derived Mϕ (1 × 10^6^) were transfected with siMNSFβ (100 nM), siRC3H1 (100 nM), siMNSFβ (100 nM) + siRC3H1 (100 nM), or NC (100 nM) in Opti-MEM without antibiotics using Lipofectamine 2000 (Thermo Fisher Scientific, USA) in accordance with the manufacturer’s protocol. After 24 h, the transfected cells were collected and used for subsequent experiments.

### Collection of Conditioned Media

Primary PRL dMϕ and Control dMϕ were cultured at a final concentration of 1 × 10^6^ cells/ml in RPMI 1640 medium supplemented with 10% FBS, 100 U/ml penicillin, and 100 µg/ml streptomycin (Gibco) for 48 h. The supernatants of the cultured PRL dMϕ were collected and used as conditioned media from PRL dMϕ (PRL dMϕ CM), while those of cultured Control dMϕ were collected and used as the Control dMϕ CM. The collected CM samples were centrifuged at 12,000 *g* for 5 min at 4°C and subsequently stored at −80°C. Thp1-derived Mϕ transfected with NC or siMNSFβ were cultured in RPMI 1640 medium with 10% FBS and 0.1% β-mercaptoethanol with antibiotics (Gibco) for 24 h. The conditioned media from siMNSFβ-transfected (siMNSFβ) or NC-transfected (NC) Thp1-derived Mϕ were collected. The samples were centrifuged at 12,000 *g* for 5 min at 4°C and stored at −80°C for subsequent experiments.

### Transwell Assay

The invasive potential of HTR8/SVneo cells was assessed *in vitro* using a BD BioCoat Matrigel Invasion Chamber (BD Biosciences, Bedford, USA) as previously described (22) with modifications. Briefly, culture medium was added into a 24-well plate and transwell inserts were plated into the wells for 2 h rehydration at 37°C. Culture medium (700 μl, 1640 with 10% FBS) was added to the lower chamber of all the wells with the transwell insert. Next, cells (4 ×10^4^ cells/well) suspended in 50 μl of RPMI 1640 with 1% FBS were combined with 50 μl specific dMϕ-CM sample, including FC-CM, Mock, Control dMϕ-CM, PRL dMϕ-CM, Control dMϕ-CM plus 10 ng/ml TNFα (Peprotech, USA), PRL dMϕ-CM plus 20 µg/ml anti-TNFα (Proteintech, USA), Mock plus 20 µg/ml anti-TNFα (Proteintech), siMNSFβ-Mϕ CM, NC-Mϕ CM, and siMNSFβ-Mϕ CM plus 10 ng/ml TNFα (Peprotech, USA), and the cell solutions were seeded in the upper chamber over the Matrigel matrix in a total volume of 100 μl. After incubating for 28 h, the noninvaded cells on the upper surface of the membrane were removed using a cotton swab. Membranes were then fixed with 4% paraformaldehyde, stained with 0.1% crystal violet (Sangon Biotech, Co., Ltd. Shanghai, China), and washed with ddH_2_O. The stained cells were counted by ImageJ software.

### Immunoprecipitation (IP)

The *in vivo* binding of MNSFβ to RC3H1 was assayed by IP. Thp1-derived Mϕ were harvested in cold phosphate-buffered saline (PBS) and washed with PBS. Then, the cell pellets were suspended in lysis buffer (50 mM Tris-HCl, 120 mM NaCl, 1% Nonidet P-40, and protease inhibitor) and incubated on ice for 30 min. After centrifugation of the cell lysates (15 min, 12,000 *g*, 4°C), supernatant samples were used for IP. Samples containing total protein extract (1 mg of protein), 4 µg of anti-MNSFβ antibody, anti-RC3H1 antibody or normal rabbit IgG, and 40 µl of protein A/G beads (50% slurry, Santa Cruz, SC-2003) were incubated at 4°C overnight with agitation. The beads were washed six times with wash buffer A (20 mM Tris-HCl, 1 mM EDTA, 900 mM NaCl, and 1% Nonidet P-40), and then washed once with wash buffer B with 100 mM NaCl before the elution (95°C, 5 min) of the bound proteins with gel-loading buffer.

### Enzyme-Linked Immunosorbent Assays (ELISAs)

The conditioned media (CM) from primary dMϕ isolated from PRL patients (PRL dMϕ) or Control women (Control dMϕ) were collected, and the TNFα levels were measured by ELISA using a commercial sandwich ELISA kit according to the manufacturer’s protocol (Quanticyto, China). The absorbance was read using an Infinite 200 Pro M Plex (TECAN). The absorbance readings were taken at 450 nm. A standard curve of TNFα was simultaneously analyzed in every plate using the dilution buffer provided by the manufacturer, and the TNFα concentrations in the samples were calculated based on the standard curve and dilution factor.

### Statistical analysis

We established biological replicates during the processing of decidual tissues from each PRL patient or normal pregnant woman. All the experiments were repeated at least three independent times, and all the values are presented as the mean ± SD. All the statistical analyses were conducted with GraphPad Prism Version 6.0. Statistical analysis was carried out by two-sided Student’s *t*-test, and differences were considered significant at *p* < 0.05.

## Results

### Distribution of MNSFβ in Decidual Macrophages at the Human Maternal–Fetal Interface

**Figure 1:**
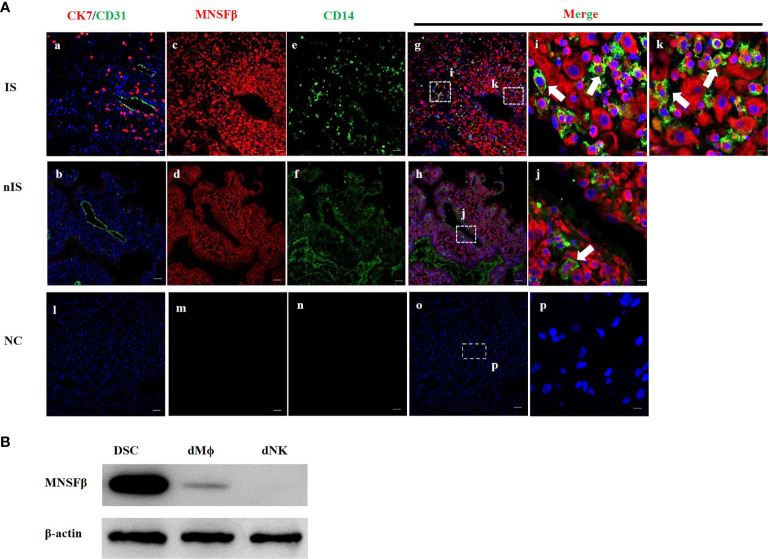
Expression of MNSFβ at the maternal–fetal interface. **(A)** Distribution of MNSFβ expression in human decidual tissues of a physically normal pregnant woman in the first trimester (8W) as determined by immunofluorescent staining analysis. **(a, b)** Staining of CK7 (red, marker of trophoblast cells) and CD31 (green, marker of endothelial cells) to confirm the embryo implantation site (IS) and nonimplantation site (nIS), and the cell nucleus was stained blue. **(c, d)** Staining of MNSFβ (red). **(e, f)** Staining of CD14 (green, marker of macrophages). **(g, h)** Staining of MNSFβ (red) and CD14 (green), and the nucleus was stained blue. **(i, k)** Magnification of images in panel **(g)**, **(j)** Magnification of images in panel **(h)**. **(l–p)** NC (negative control) of CK7/CD31, MNSFβ, and CD14. White arrow indicates typical positive cells. Scale bars of a–h represent 100 μm; scale bars of i–k represent 10 μm. **(B)** Detection of the MNSFb protein expression in human decidual stromal cells (DSCs), decidual macrophages (dMФ), and decidual NK cells (dNKs) of early pregnancy by Western blot analysis.

As the expression of MNSFβ in human dMϕ has not yet been reported, immunofluorescent staining analysis was carried out to determine the localization of the MNSFβ protein in human decidual tissues during early pregnancy (8W). In addition to decidual stromal cells (DSCs), CK7, CD31, and CD14 protein signals were used as markers of trophoblast cells, endothelial cells, and macrophages, respectively. CK7-positive staining indicated the implantation site (IS), whereas CK7-negative staining indicated the nonimplantation site (nIS). The results showed that the MNSFβ protein signals were widely distributed in human decidual tissues, including decidual macrophages, during the first trimester (**Figure 1A**). In addition, the protein expression of MNSFβ in human DSCs and dMϕ during early pregnancy was also detected by Western blotting analysis (**Figure 1B**).

### Increased MNSFβ Expression in Decidual Macrophages Isolated From RPL Patients

**Figure 2:**
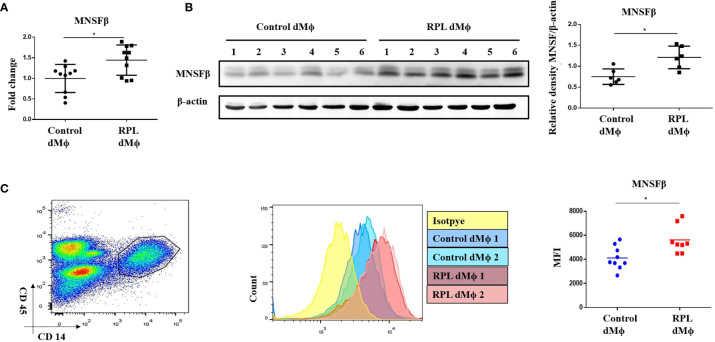
Alteration of the MNSFβ expression in human dMϕ from RPL patients in the first trimester. **(A)** MNSFβ mRNA expression levels in dMϕ from Control women (*n* = 10) and RPL patients (*n* = 10) as detected by real-time RT-PCR analysis (normalized to the mRNA expression of Gapdh). **(B)** MNSFβ protein expression levels in dMf from Control women (*n* = 6) and RPL patients (*n* = 6) as detected by Western blotting analysis (normalized to the expression of b-actin). Left panel: Representative images of Western blotting assay; right panel: the relative density of MNSFb/b-actin. **(C)** MNSFβ protein expression levels in dMФ from Control women (*n* = 9) and RPL patients (*n* = 8) as detected by flow cytometry. Left panel: flow cytometry analysis of dMФ with antibodies against CD45 and CD14; middle panel: representative images of the flow cytometry assay; right panel: the mean fluorescence intensity (MFI) of MNSFβ as detected by flow cytometry. (Control dMФ: dMФ isolated from decidual tissues of normal women in early pregnant; RPL dMФ: dMФ isolated from decidual tissues of RPL patients, *p < 0.05).

In our previous studies, the MNSFβ expression levels in both the decidual and villus tissues from RPL patients were observed to be significantly decreased compared to those in the tissues from normal pregnant women (16, 17); thus, we isolated dMϕ from RPL patients (RPL dMϕ) and normal pregnant women (Control dMϕ) with ~90% purity (**Supplementary Figure S1A**) by using CD14 and CD45 as biomarkers (19), and we hypothesized that the MNSFβ expression level in RPL dMϕ would also be reduced. Unexpectedly, the RT-PCR (**Figure 2A**) and Western blotting (**Figure 2B**) results showed that the MNSFβ expression level in RPL dMϕ was significantly increased compared to that in Control dMϕ. The upregulated MNSFβ expression in RPL dMϕ was further confirmed by flow cytometry (**Figure 2C**). However, the MNSFβ expression level in the total cells isolated from the decidual tissues from RPL patients was obviously reduced compared to that in the total cells isolated from the decidual tissues from Control women (**Supplementary Figure S1B**).

### Proportion of CD11c^high^ dMφ Was Increased While That of CD11c^low^ dMφ Was Decreased in RPL Patients

**Figure 3:**
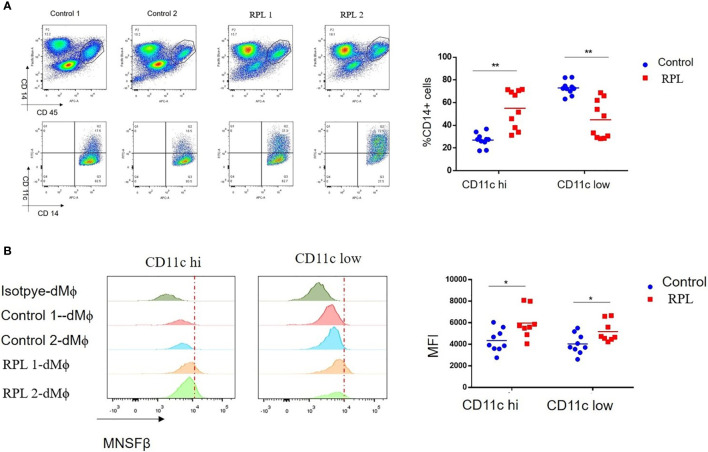
Changes in the proportion of the CD11c high and CD11c low subsets in human dMϕ from RPL patients. **(A)** Proportion of the CD11c high (CD11c hi) and CD11c low (CD11c low) subsets in the dMϕ of Control women (*n* = 9) and RPL patients (*n* = 8) as detected by flow cytometry. Left panel: flow cytometry analysis of dMϕ with antibodies against CD45 plus CD14 and CD14 plus CD11c. Right panel: Proportions of CD11c hi and CD11c low dMϕ. **(B)** MNSFβ protein expression levels in CD11c hi and CD11c low dMϕ from Control women (*n* = 9) and RPL patients (*n* = 8) as detected by flow cytometry. Left panel: Representative images of the flow cytometry assay; right panel: the mean fluorescence intensity (MFI) of MNSFβ as detected by flow cytometry. (Control: dMϕ isolated from decidual tissues of normal women in early pregnancy; RPL: dMϕ isolated from decidual tissues of RPL patients, *p < 0.05, **p < 0.01).

Macrophages are usually classified into M1 and M2 subtypes; however, human dMϕ could not be clustered by M1 and M2 markers (18). It has been reported that macrophages in normal first-trimester decidual tissues could be categorized into CD11c^high^ (~20%) and CD11c^low^ (~80%) subsets (18, 19). In the present study, we separated CD11c^high^ dMϕ (CD11c hi) and CD11c^low^ dMϕ (CD11c low) by flow cytometry and found that the proportions of the CD11c hi and CD11c low subsets in Control women were 27.0% ± 6.4% and 73.0% ± 6.4%, respectively, whereas those of the CD11c hi and CD11c low subsets in RPL patients were significantly increased to 58.4 ± 16.2% and dramatically decreased to 41.6 ± 16.2%, respectively (**Figure 3A**). No significant difference in the MNSFβ expression level was observed between the CD11c hi subset and CD11c low subset; however, the MNSFβ expression levels in the CD11c hi and CD11c low subsets from RPL patients were significantly increased compared to that of Control women (**Figure 3B**).

### Expression and Secretion of TNFα Were Increased in dMϕ From RPL Patients

**Figure 4:**
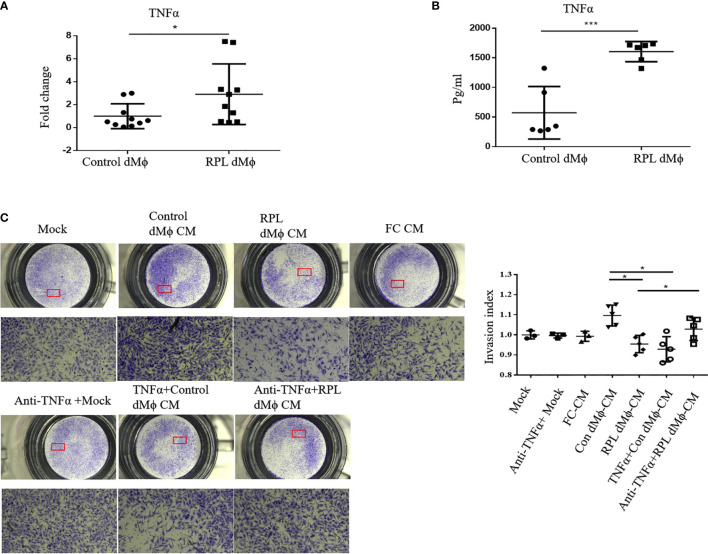
Changes in the expression and secretion levels of TNFα in dMϕ from RPL patients. **(A)** TNFα mRNA expression levels in dMϕ from Control women (*n* = 10) and RPL patients (*n* = 10) as detected by real-time RT-PCR analysis (normalized to the mRNA expression of Gapdh). **(B)** Concentration of TNFα in the conditioned media (CM) of cultured primary dMϕ from Control women (*n* = 6) and RPL patients (*n* = 6) as detected by ELISA. **(C)** Invasion of HTR8/SVneo cells as detected by Transwell assay. Left: Representative images of the Transwell assay; Right: quantifications of the Transwell assay by the invasion index [invasion index = X (invaded cells number)/Mock (invaded cells number)]. (Control dMϕ: dMϕ from normal women in early pregnancy; RPL dMϕ: dMϕ from RPL patients; Mock: treated with medium without cells; FC CM: treated with CM of human fibroblasts; Control dMФ CM: treated with CM of Control dMФ; RPL dMФ CM: treated with CM of RPL dMФ; TNFα + Control dMФ CM: treated with CM of Control dMФ plus TNFα; anti-TNFα+RPL dMФ CM: treated with CM of RPL dMФ plus anti-TNFα antibody; *p < 0.05; ***p < 0.001).

As it has been reported that MNSFβ inhibits TNFα expression in murine macrophages (12), we reasonably thought that the TNFα expression level might be decreased in RPL dMϕ because MNSFβ expression was upregulated. However, the RT-PCR results showed that the TNFα expression level in cultured primary dMϕ from RPL patients (RPL dMϕ) was significantly enhanced compared to that in cultured primary dMϕ from normal pregnant women (Control dMϕ) (**Figure 4A**). The TNFα concentration in the conditioned media of RPL dMϕ (RPL dMϕ CM) was also higher than that in the conditioned media of Control dMϕ (Control dMϕ CM) (**Figure 4B**), indicating that TNFα production and secretion levels were increased in the dMϕ from RPL patients.

### Decidual Macrophages of RPL Patients Inhibited HTR8/SVneo Cell Invasion Partially Through TNFα

TNFα could inhibit the invasion of HTR8/SVneo cells (22), and we found that the level of TNFα secretion by RPL dMϕ was increased; thus, we also examined the effect of the conditioned media of dMϕ on the invasion of HTR8/SVneo cells. The Transwell assay results showed that the invasion of HTR8/SVneo cells treated with RPL dMϕ CM was significantly reduced compared with that of cells treated with Control dMϕ CM (**Figure 4C**), indicating that RPL dMϕ might inhibit the invasion of EVTs in a paracrine manner. More interestingly, the invasion of HTR8/SVneo cells was inhibited by Control dMϕ CM plus TNFα (TNFα+Control dMϕ CM), whereas the inhibitory effect of RPL dMϕ CM on the invasion of HTR8/SVneo cells could be restored by the addition of an anti-TNFα antibody (anti-TNFα+ RPL dMϕ CM) (**Figure 4C**); these results suggested that dMϕ might regulate the invasion of EVTs by secreting TNFα.

### Knockdown of MNSFβ in Mϕ Resulted in Reduced TNFα Production and Enhanced HTR8/SVneo Cell Invasion

**Figure 5:**
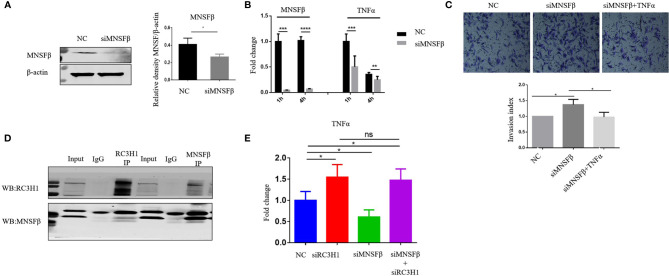
MNSFβ promoted the expression and secretion of TNFα by binding to RC3H1 in human macrophages. **(A)** MNSFβ protein expression level in human Thp1-derived Mϕ as detected by Western blotting analysis (normalized to the expression of β-actin). Left panel: representative images of Western blotting assay. Right panel: the relative density of MNSFβ/β-actin. **(B)** MNSFβ and TNFα mRNA expression levels in Thp1-derived Mϕ as detected by RT-PCR analysis (normalized to the mRNA expression of Gapdh). **(C)** Invasive activity of HTR8/SVneo cells detected by transwell assay. Upper: representative images of the Transwell assay; lower: quantification of the Transwell assay by the invasion index [invasion index = X (invaded cells number)/NC (invaded cells number)]. **(D)** Interaction between MNSFβ and RC3H1 as validated by co-IP assay. **(E)** TNFα mRNA expression level in Thp1-derived Mϕ detected by RT-PCR analysis (normalized to the mRNA expression of Gapdh). (NC: Thp1-derived Mϕ transfected with NC-siRNA or HTR8/SVneo cells treated with the CM of NC Mϕ; siMNSFβ: Thp1-derived Mϕ transfected with MNSFβ-specific siRNA or HTR8/SVneo cells treated with the CM of siMNSFβ Mϕ; siMNSFβ + TNFα: HTR8/SVneo cells treated with the CM of siMNSFβ Mϕ plus TNFα; siRC3H1: Thp1-derived Mϕ transfected with RC3H1 specific siRNA; siMNSFβ + siRC3H1: Thp1-derived Mϕ transfected with MNSFβ-specific siRNA and RC3H1-specific siRNA; ns p > 0.05, *p < 0.05; ***p < 0.001; ****p< 0.0001).

The results of the experiments mentioned above suggested that MNSFβ expression in RPL dMϕ was increased, and RPL dMϕ might inhibit the invasion of EVTs *via* increased TNFα secretion, indicating a potential positive correlation between MNSFβ expression levels and TNFα production levels in human dMϕ. Thus, we observed the effect of downregulated MNSFβ expression on the TNFα production induced by LPS in human Thp1-derived Mϕ; a suitable *in vitro* model to investigate the Mϕ functions (23). The results showed that in Thp1-derived Mϕ, the MNSFβ expression level could be significantly knocked down by transfection with a specific siRNA, and the TNFα expression level was also decreased in MNSFβ-knockdown Mϕ (**Figures 5A, B**). Furthermore, the invasion of HTR8/SVneo cells was enhanced by treatment with the CM of MNSFβ-knockdown Mϕ (siMNSFβ), and this stimulatory effect could be eliminated by the addition of TNFα (**Figure 5C**); these results further indicated that dMϕ could inhibit the invasion of EVTs by secreting TNFα.

### MNSFβ Interacted With RC3H1 to Regulate TNFα Expression in Human Macrophages

To explore the molecular mechanism underlying the positive correlation between MNSFβ expression and TNFα expression in human macrophages, we searched for and predict proteins that could potentially interact with MNSFβ through BioGRID (https://thebiogrid.org/), and RC3H1 was identified as a candidate (**Supplementary Figure S3**). Given that RC3H1 could inhibit TNFα expression (24), we hypothesized that MNSFβ might promote TNFα production by weakening the inhibitory effect of RC3H1 on TNFα expression. Thus, we investigated the interaction between MNSFβ and RC3H1 in human macrophages by co-IP assay. The results showed direct binding between the MNSFβ and RC3H1 proteins in Thp1-derived Mϕ (**Figure 5D**). Then, we knocked down the expression of MNSFβ or RC3H1 in Thp1-derived Mϕ and found that the TNFα expression level was increased in RC3H1-knockdown (siRC3H1) cells but decreased in MNSFβ-knockdown (siMNSF) cells (**Figure 5E**). These results suggested that MNSFβ might promote the expression of TNFα by binding to RC3H1.

### Protein Level of RC3H1 Was Decreased in dMϕ From RPL Patients

**Figure 6:**
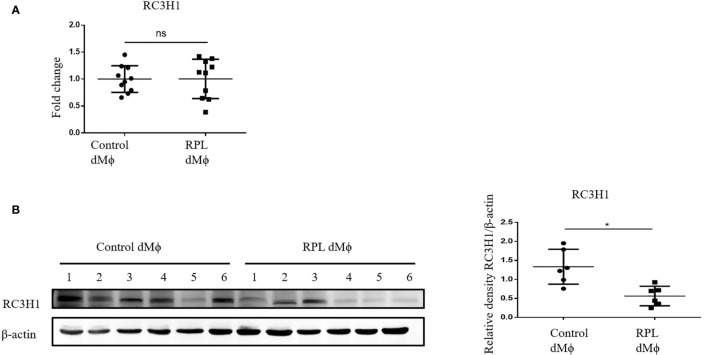
Changes in the RC3H1 protein expression level of dMϕ from RPL patients. **(A)** RC3H1 mRNA expression level in dMϕ from Control women (*n* = 10) and RPL patients (*n* = 10) as detected by RT-PCR analysis (normalized to the mRNA expression of Gapdh). **(B)** RC3H1 protein expression level in dMϕ from Control women (*n* = 6) and RPL patients (*n* = 6) as detected by Western blotting analysis (normalized to the expression of β-actin). Left panel: representative images of Western blotting assay; right panel: the relative density of RC3H1/β-actin. (Control dMϕ: dMϕ isolated from decidual tissues from normal women in early pregnancy; RPL dMϕ: dMϕ isolated from decidual tissues of RPL patients, ns p > 0.05, *p < 0.05).

We also detected the RC3H1 expression level in primary human decidual macrophages. As we hypothesized, the protein expression of RC3H1 was decreased in RPL dMϕ compared with Control dMϕ (**Figure 6B**). However, the RC3H1 mRNA expression level was not significantly different (**Figure 6A**), indicating that MNSFβ might only affect the protein level of RC3H1.

## Discussion

**Figure 7:**
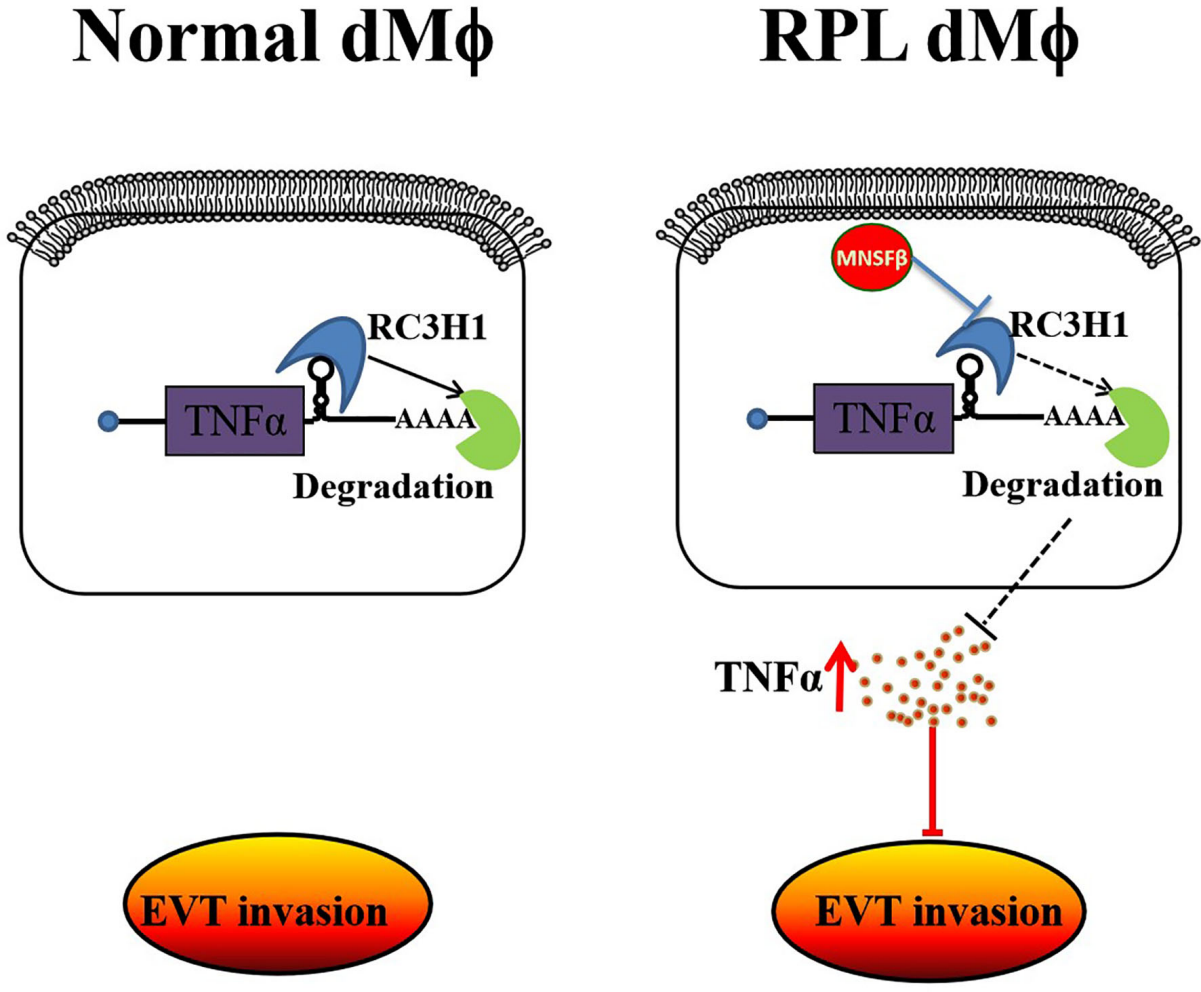
Schematic showing that MNSFβ promotes TNFα production, which inhibits the invasion of trophoblasts, by interacting with RC3H1 in RPL dMϕ; dMϕ: decidual tissue macrophages.

It was found in the present study that, at the maternal–fetal interface of RPL patients, dMϕ showed an inclination to CD11c^high^ subtype, instead of CD11c^low^ subtype, accompanied with the significantly increased productions of MNSFβ and TNFα, and the reduced production of RC3H1. MNSFβ might promote the secretion of TNFα by binding to RC3H1. Compared to dMϕ of Control women at early pregnancy, dMϕ of RPL patients could strongly inhibit the invasive activity of extravillous trophoblasts (EVTs) in a paracrine manner at least partially mediated by TNFα (**Figure 7**).

Successful establishment of mammals’ pregnancy depends on the generation of maternal–fetal immune tolerance and remodeling of the spiral arteries (25). Dysfunction in these processes has been correlated with adverse pregnancy outcomes including RPL (26). Although it has been well recognized that dMϕ participate in the immune modulation and spiral artery remodeling at the maternal–fetal interface (25, 27), the exact roles of dMϕ in these events are still not well understood.

We previously found that, MNSFβ expression was significantly decreased in both decidual and villus tissues from RPL patients (16, 17). The knockdown of MNSFβ expression could inhibit proliferation and migration of human EVTs (17), as well as proliferation (**Supplementary Table S4**) and decidualization (**Supplementary Figure S4**) of human endometrial stromal cells (ESCs) (unpublished data). However, whether MNSFβ participates in the regulation of decidual immune cells, especially dMϕ, has been unknown.

Given decidual MNSFβ expression was reduced in RPL patients, and the secreted form of MNSFβ was identified as an immune suppressor, it was reasonable to hypothesize that MNSFβ expression in dMϕ of RPL patients might well be decreased to destroy the immunotolerance at the maternal–fetal interface. However, unexpectedly, in this study, it was observed that, MNSFβ expression in RPL dMϕ was obviously increased (**Figure 2**), whereas its expression in total decidual cells from RPL patients was significantly decreased as expected (**Supplementary Figure S1C**). These data suggested that MNSFβ might play different roles in various cells at the maternal–fetal interface.

Thus, we hypothesized that MNSFβ may be involved in the regulation of cell proliferation and invasion of EVTs, cell proliferation and decidualization of ESCs, and cell differentiation and secretion of DICs. Therefore, the decreased MNSFβ expression in EVTs and DSCs might lead to pregnancy loss by interfering with the invasion of EVTs and generation of decidua; however, in dMϕ, the abnormally increased MNSFβ expression might disrupt the immune homeostasis of the local microenvironment and result in pregnancy failure.

Macrophages are usually classified into the M1 subtype, a proinflammatory phenotype, or the M2 subtype, an anti-inflammatory phenotype (28). Enhanced M1 polarization, characterized by increased TNFα expression, is associated with RPL or preeclampisa (29, 30). However, due to the lack of appropriate biomarkers, we were unable to separate M1 and M2 dMϕ from the decidual tissues at a reasonably high purity. Fortunately, it was reported that two distinct subtypes of dMϕ, namely, CD11c hi and CD11c low, could be specifically separated by the cell surface marker CD11c (18, 19). Although they secrete both pro-inflammatory and anti-inflammatory cytokines, CD11c hi dMϕ are thought to be involved in lipid metabolism and inflammation, whereas CD11c low dMϕ are related to the extracellular matrix formation and tissue growth (18). However, whether the alteration in CD11c dMϕ polarization associates with RPL is unclear.

It was reported that, CD11c hi dMϕ are localized close to EVTs (19), and TNFα is highly expressed in CD11c hi Mϕ (31). TNFα could inhibit the invasiveness of EVTs (22), and increased decidual and peripheral TNFα levels were observed in RPL patients (32). Interestingly, we found that the proportion of CD11c hi dMϕ was significantly raised in RPL patients (**Figure 3**), and the production and secretion level of TNFα was obviously increased in dMϕ from RPL patients (**Figure 4**). In addition, compared to dMϕ from normal women early in pregnancy, dMϕ from RPL patients exerted a stronger inhibitory effect on the invasion of EVTs in a paracrine manner, and this effect could be effectively reversed by anti-TNFα antibody (**Figure 4**). These results indicated that the appropriate CD11c dMϕ polarization was important for the micro-homeostasis at the maternal–fetal interface.

In this study, we found that knockdown of MNSFβ led to the decreased TNFα expression in human Mϕ. This phenomenon conflicted with previous reports that MNSFβ inhibits TNFα production in LPS-activated murine Mϕ (12). We hypothesized that MNSFβ might regulate the production of TNFα by different molecular pathways in different cells. Although several molecules, such as Bcl-G (10), endocytosin II (11), HSPA8 (33), and Hsp60 (34), have been identified to bind with MNSFβ in murine Mϕ, none of them could explain the promoting effect of MNSFβ on promoting TNFα expression. Thus, we identified candidates in the BioGRID database (**Supplementary Figure S3**) and found that MNSFβ potentially binds to RC3H1, which could inhibit the expression of TNFα by degrading its mRNA (24, 35), and as an immune regulator, RC3H1 is involved in T-cell activation (36).

We supposed that, as a ubiquitin-like protein, MNSFβ might eliminate the inhibitory effect of RC3H1 on TNFα expression by degrading the RC3H1 protein. Consistently, the direct interaction between MNSFβ and RC3H1 was subsequently confirmed, and knockdown of RC3H1 expression in human Mϕ could lead to increased TNFα expression (**Figure 5**). More encouragingly, a significantly decreased level of the RC3H1 protein was detected in the dMϕ from RPL patients (**Figure 6B**), suggesting that the increased MNSFβ expression in dMϕ might promote the production and secretion of TNFα by binding to RC3H1, and the secreted TNFα could inhibit the invasion of EVTs, resulting in early pregnancy loss. Furthermore, we also observed that the secretive level of MNSFβ protein was simultaneously increased in dMϕ from RPL patients (**Supplementary Figure S5**). The increased secretion of MNSFβ in dMϕ might have synergistic effects with secreted TNFα on invasive activities of EVTs during early pregnancy, but need further investigation.

In addition, although it was found that dMϕ had no effect on the invasion of primary EVTs (37), it was also reported that LPS-stimulated peripheral blood monocytes could inhibit HTR-8/SVneo cell invasion by the secretion of TNFα (38), and M2 macrophages showed an enhanced promotion effect on trophoblast cell motility (39); these suggest that the dMϕ might inhibit EVTs invasion in early pregnancy. However, in this study, we found that the dMϕ from normal women in early pregnancy exerted a stimulatory effect on HTR-8/SVneo cell motility (**Figure 4C**). DMϕ not only secrete TNF-α and IL-10, both of which could inhibit trophoblast motility, but also IL-1β and IL-8, both of which could stimulate trophoblast motility (40, 41); enhanced EVT motility would promote their invasion into endometrial tissue, whereas reduced EVT motility would be required to prevent their excessive invasion; thus, we hypothesized that dMϕ might promote the motility of EVTs in the early stage of the first trimester, but inhibit their motility in the late stage of the first trimester to guarantee the appropriate invasion of EVTs into maternal uterine tissues. Such an exquisite and complex regulation of EVTs invasion by dMϕ is worthy of further investigation.

It should be noted that the decidual tissue samples from RPL patients were collected after the death of the fetus; thus, the differences in MNSFβ expression and the ratio of the CD11c hi/CD11c low subsets might be consequences of abortion instead of its pathogenic causes. In addition, we fully understood that, since MNSFβ expression was increased in RPL dMϕ, we should observe the effect of upregulated MNSFβ expression on the functions of macrophages. However, although we have successfully established the MNSFβ overexpression HTR8/SVneo cell model (17), we failed to establish MNSFβ-overexpressing cell models in both Thp1-derived Mϕ and T-HESCs for unknown reasons. Thus, in future investigations, we should establish a reasonably large prospective cohort of women in early pregnancy as well as a macrophage-specific MNSFβ gene knock-in mouse model to systematically explore the roles of MNSFβ in the pathogenesis of RPL.

In summary, it was found in this study that the MNSFβ expression level and the proportion of CD11c hi cells among dMϕ, as well as the TNFα production and secretion level in dMϕ, were significantly increased in RPL patients. *In vitro*, RPL dMϕ showed a TNFα-mediated inhibitory effect on the invasion of HTR8/SVneo cells in a paracrine manner. In human Mϕ, MNSFβ could promote the TNFα production by binding to RC3H1. Furthermore, the RC3H1 protein level in RPL dMϕ was significantly decreased. These data suggested that MNSFβ played important roles in human dMϕ at least partially *via* the RC3H1–TNFα pathway. The abnormally increased MNSFβ expression in human dMϕ might lead to early pregnancy loss by inducing the polarization of dMϕ toward a proinflammatory phenotype and promoting the secretion of TNFα at the maternal–fetal interface.

## Data Availability Statement

The original contributions presented in the study are included in the article/**Supplementary Material**. Further inquiries can be directed to the corresponding authors.

## Ethics Statement

The studies involving human participants were reviewed and approved by The Medical Ethics Committees of The Second Hospital of Tianjin Medical University and The Medical Ethical Committee, Shanghai Institute for Biomedical and Pharmaceutical Technologies (former Shanghai Institute of Planned Parenthood Research) (Seal). The patients/participants provided their written informed consent to participate in this study.

## Author Contributions

Y-LW and JW designed and supervised the study, integrated the data, and revised the manuscript. X-XZ, Y-PH and LY performed experiments of the flow cytometry, WB, qPCR, co-IP, cell culture, and transwell assay. YG contributed to the collection of clinical samples. QY participated in immunofluorescence staining and confocal microscopy. W-WG contributed to data analysis. All authors contributed to the article and approved the submitted version.

## Funding

This work was supported by grants from the National Key Research and Development Program of China (2018YFC1002801), the Natural Science Foundation of China (81671459, 81730040, 81973327, and 82001640), and Shanghai S&T Innovation Plan (18140902901).

## Conflict of Interest

The authors declare that the research was conducted in the absence of any commercial or financial relationships that could be construed as a potential conflict of interest.

## Publisher’s Note

All claims expressed in this article are solely those of the authors and do not necessarily represent those of their affiliated organizations, or those of the publisher, the editors and the reviewers. Any product that may be evaluated in this article, or claim that may be made by its manufacturer, is not guaranteed or endorsed by the publisher.

## Supplementary Material

Supplementary Figure S1 Changes in the MNSFβ expression level in total decidual cells from RPL patients. **(A)** Purity of dMϕ isolated from decidual tissues of a normal woman in early pregnancy. **(B)** MNSFβ expression level in total decidual cells from Control women (n=9) and RPL patients (n=8) as detected by flow cytometry. Left panel: Representative images of the flow cytometry assay; right panel: the mean fluorescence intensity (MFI) of MNSFβ as detected by flow cytometry. (Control dMϕ: dMϕ isolated from decidual tissues from normal women in early pregnancy; Control: total decidual cells isolated from decidual tissues of normal women in early pregnancy; RPL: total decidual cells isolated from decidual tissues from RPL patients, *P < 0.05).

Supplementary Figure S2 Thp1-derived Mϕ identification. Thp1 cells were incubated with PMA for 24 hrs, and the expression of markers (CD11b and SPI1) of macrophage maturation was detected by RT-PCR. The data are shown as the mean ± SD. **P < 0.01; ***P < 0.001.

Supplementary Figure S3 Protein candidates that potentially interact with MNSFβ were predicted by searching the BioGRID database (https://thebiogrid.org). FAU: MNSFβ.

Supplementary Figure S4 Detection of MNSFβ protein expression levels during the *in vitro* decidualization of the human endometrial stromal cell line T-HESC (induced by estrogen, progesterone and cAMP) by Western blotting analysis.

Supplementary Figure S5 Levels of secretive MNSFβ (sMNSFβ) in the conditioned media of cultured primary dMϕ from Control women (n=6) and RPL patients (n=6) as detected by dot blotting assay. Upper: Representative images of dot blotting assay; Below: the relative density of sMNSFb/ul. CM: completed medium; Control dMϕ: dMϕ isolated from decidual tissues of normal women in early pregnancy; RPL dMϕ: dMϕ isolated from decidual tissues of RPL patients, **P < 0.01).

Supplementary Table S1 Clinical Characteristic of Recurrent Pregnancy Loss (RPL) Patients and Normal Pregnant (Control) Women Whose Decidua Tissues Were Used in This Study.

Supplementary Table S2 Sequence of Primers used in Real-time RT-PCR.

Supplementary Table S3 Information of Antibodies Used in This Study.

Supplementary Table S4 Proliferation of T-HESCs by Cell Viability Analysis.
