# Supplementary figures and images for: MNSFβ Regulates TNFα Production by Interacting with RC3H1 in Human Macrophages, and Dysfunction of MNSFβ in Decidual Macrophages Is Associated With Recurrent Pregnancy Loss
Source: Front Immunol. 2021 Sep 13;12:691908. doi: 10.3389/fimmu.2021.691908 (PMC8473736; doi:10.3389/fimmu.2021.691908)

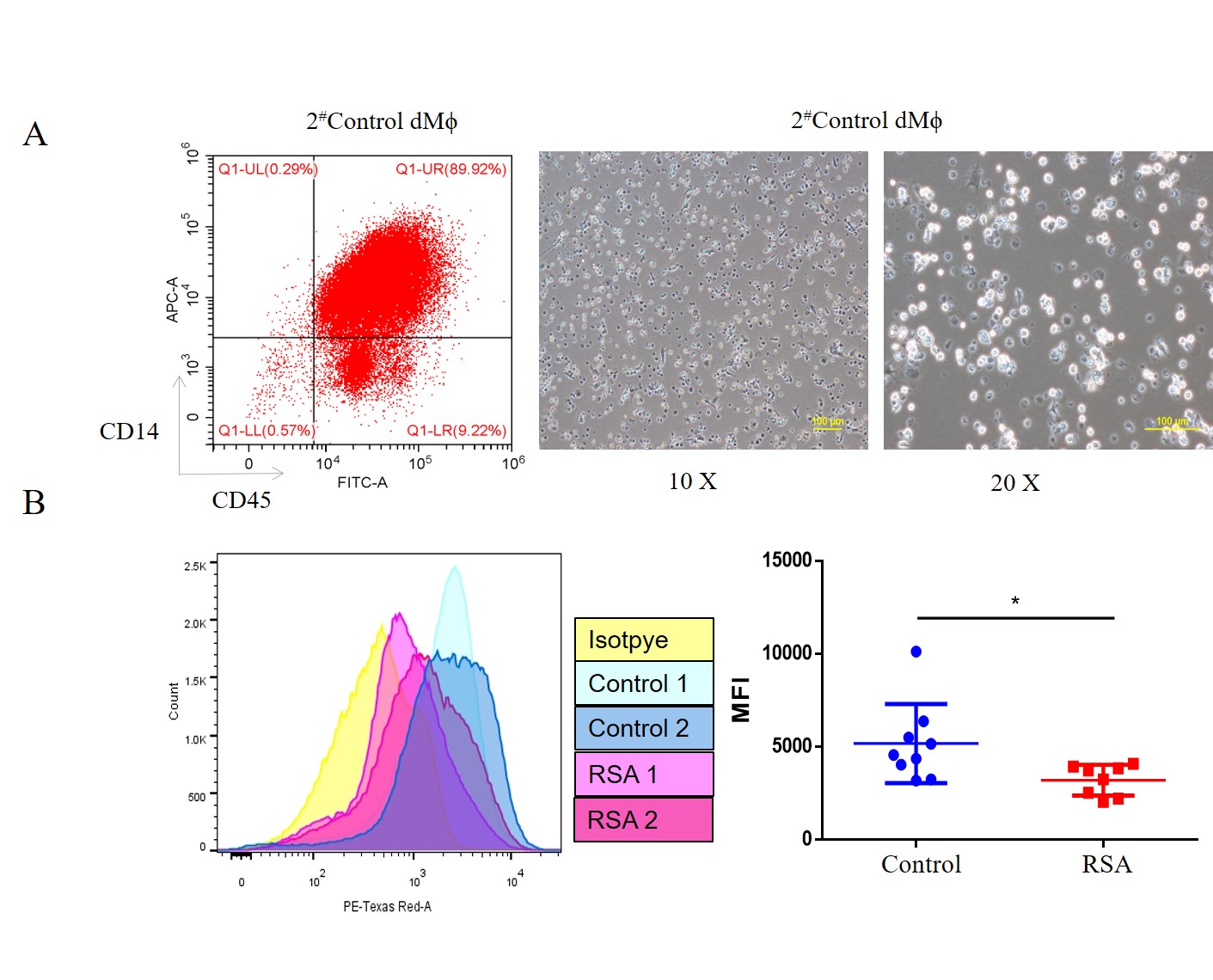

Supplement: Supplementary Figure S1 — Changes in the MNSFβ expression level in total decidual cells from RPL patients. (A) Purity of dMϕ isolated from decidual tissues of a normal woman in early pregnancy. (B) MNSFβ expression level in total decidual cells from Control women (n=9) and RPL patients (n=8) as detected by flow cytometry. Left panel: Representative images of the flow cytometry assay; right panel: the mean fluorescence intensity (MFI) of MNSFβ as detected by flow cytometry. (Control dMϕ: dMϕ isolated from decidual tissues from normal women in early pregnancy; Control: total decidual cells isolated from decidual tissues of normal women in early pregnancy; RPL: total decidual cells isolated from decidual tissues from RPL patients, *P < 0.05). [file Image_1.jpeg]

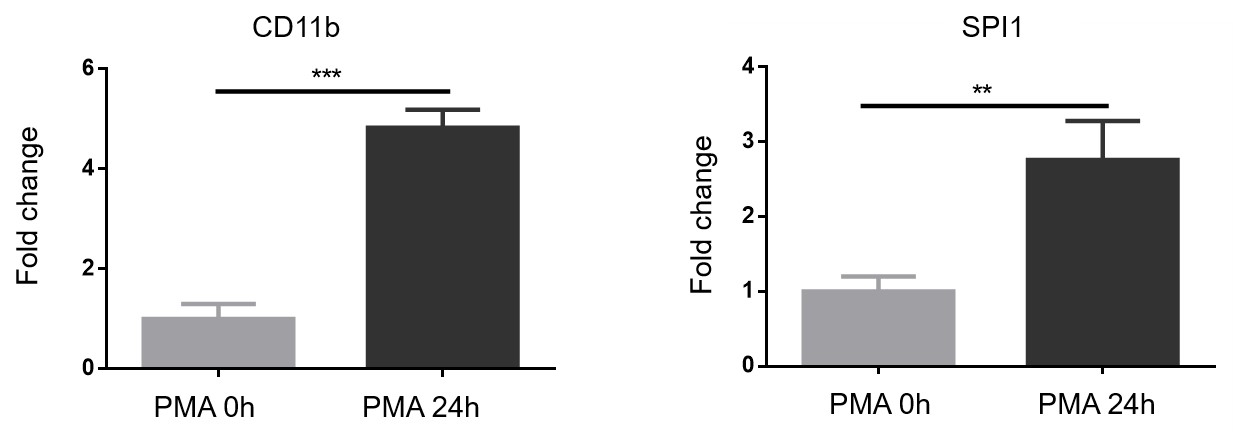

Supplement: Supplementary Figure S2 — Thp1-derived Mϕ identification. Thp1 cells were incubated with PMA for 24 hrs, and the expression of markers (CD11b and SPI1) of macrophage maturation was detected by RT-PCR. The data are shown as the mean ± SD. **P < 0.01; ***P < 0.001. [file Image_2.jpeg]

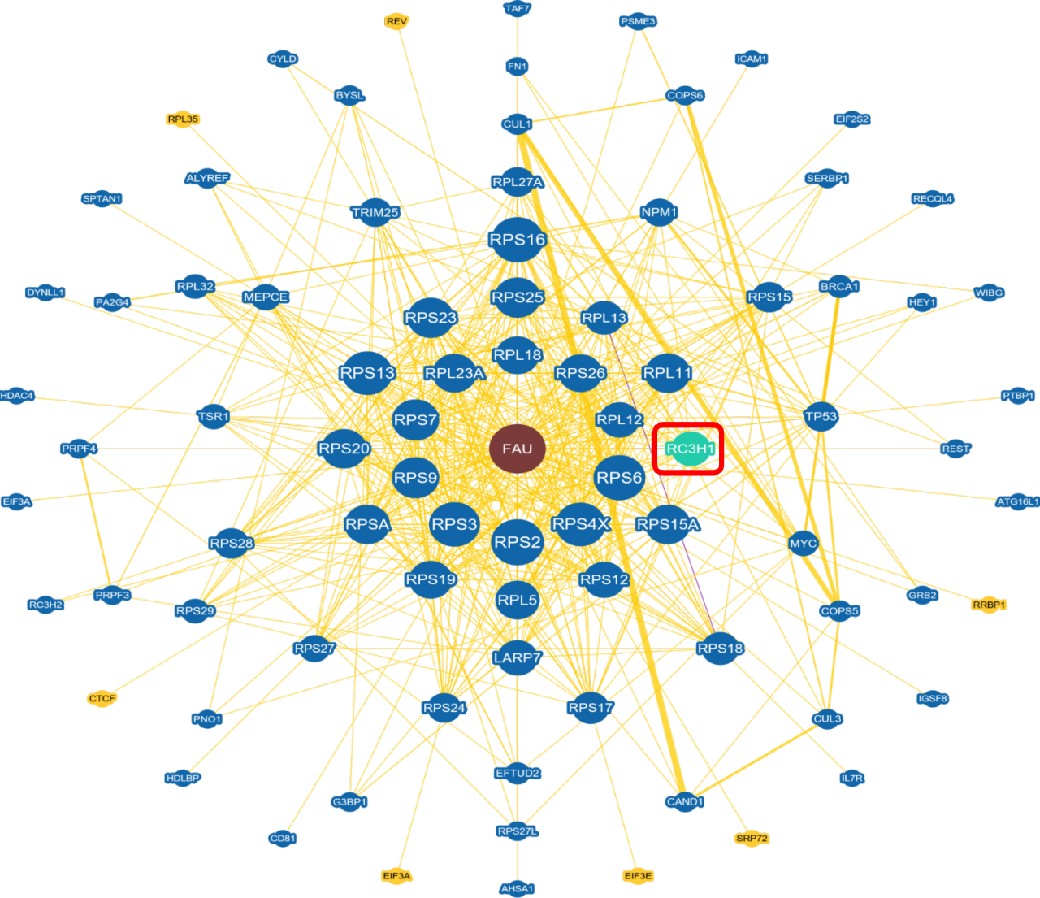

Supplement: Supplementary Figure S3 — Protein candidates that potentially interact with MNSFβ were predicted by searching the BioGRID database (https://thebiogrid.org). FAU: MNSFβ. [file Image_3.jpeg]

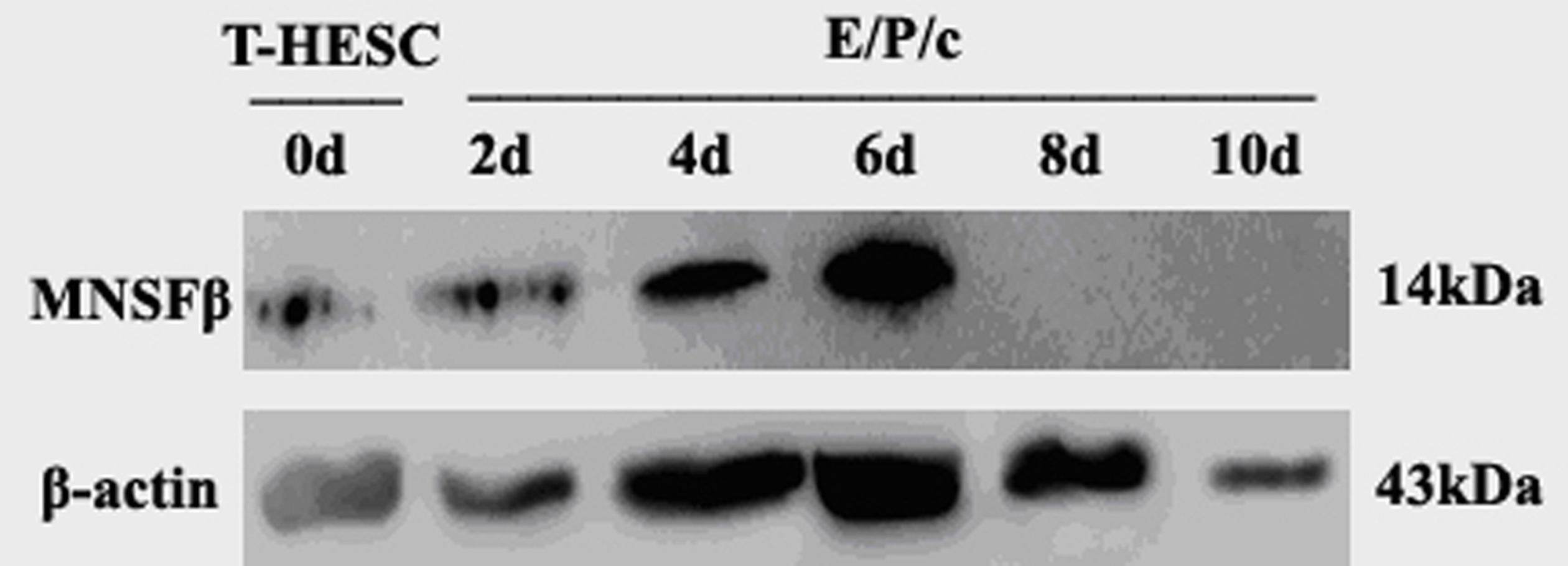

Supplement: Supplementary Figure S4 — Detection of MNSFβ protein expression levels during the in vitro decidualization of the human endometrial stromal cell line T-HESC (induced by estrogen, progesterone and cAMP) by Western blotting analysis. [file Image_4.jpeg]

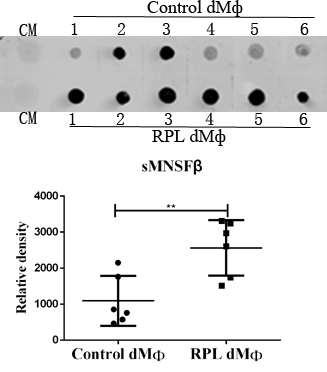

Supplement: Supplementary Figure S5 — Levels of secretive MNSFβ (sMNSFβ) in the conditioned media of cultured primary dMϕ from Control women (n=6) and RPL patients (n=6) as detected by dot blotting assay. Upper: Representative images of dot blotting assay; Below: the relative density of sMNSFb/ul. CM: completed medium; Control dMϕ: dMϕ isolated from decidual tissues of normal women in early pregnancy; RPL dMϕ: dMϕ isolated from decidual tissues of RPL patients, **P < 0.01). [file Image_5.tif]
