# Supplementary material for: MNSFβ Regulates TNFα Production by Interacting with RC3H1 in Human Macrophages, and Dysfunction of MNSFβ in Decidual Macrophages Is Associated With Recurrent Pregnancy Loss
Source: Front Immunol. 2021 Sep 13;12:691908. doi: 10.3389/fimmu.2021.691908 (PMC8473736; doi:10.3389/fimmu.2021.691908)
Supplement: Supplementary Table S1 — Clinical Characteristic of Recurrent Pregnancy Loss (RPL) Patients and Normal Pregnant (Control) Women Whose Decidua Tissues Were Used in This Study. [file Table_1.pdf]

TABLE S1 | Clinical Characteristic of Recurrent Pregnancy Loss (RPL) Patients and Normal Pregnant (Control) Women Whose Decidua Tissues Were Used in This Study

Group	n	Age (years)	Gestational Week	Pregnant history	Childbearing history	Miscarriage history
Control	25	33.9 ±4.3	7.7 ±1.0	1.8 ±0.6	0.7 ±0.6	0.0 ±0.0
RPL	24	32.0 ±4.1	7.8 ±0.9	2.8 ±0.8	0.4 ±0.6	2.4 ±0.7
P value				<0.01		<0.0001
