# Supplementary material for: MNSFβ Regulates TNFα Production by Interacting with RC3H1 in Human Macrophages, and Dysfunction of MNSFβ in Decidual Macrophages Is Associated With Recurrent Pregnancy Loss
Source: Front Immunol. 2021 Sep 13;12:691908. doi: 10.3389/fimmu.2021.691908 (PMC8473736; doi:10.3389/fimmu.2021.691908)
Supplement: Supplementary Table S2 — Sequence of Primers used in Real-time RT-PCR. [file Table_2.pdf]

TABLE S2 | Sequence of Primers used in Real-time RT-PCR

**Primer**	**Nucleotide sequence** (5′to3′)
homo MNSFβ Forward	ACTCCATCTTCGCGGTAGC
homo MNSFβ Reverse	GGAGCACGACTTGATCTTCC
homo TNFα Forward	CCTCTCTCTAATCAGCCCTCTG
homo TNFα Reverse	GAGGACCTGGGAGTAGATGAG
homo GAPDH Forward	CTGGGCTACACTGAGCACC
homo GAPDH Reverse	AAGTGGTCGTTGAGGGCAATG
homo RC3H1 Forward	TCCACAATGGACGGATTTCCT
homo RC3H1 Reverse	AACCCAAACTGATGGGCTTTC
