# Supplementary material for: MNSFβ Regulates TNFα Production by Interacting with RC3H1 in Human Macrophages, and Dysfunction of MNSFβ in Decidual Macrophages Is Associated With Recurrent Pregnancy Loss
Source: Front Immunol. 2021 Sep 13;12:691908. doi: 10.3389/fimmu.2021.691908 (PMC8473736; doi:10.3389/fimmu.2021.691908)
Supplement: Supplementary Table S3 — Information of Antibodies Used in This Study. [file Table_3.pdf]

TABLE S3 | Information of Antibodies Used in This Study

Antibodies	Catalog Number	Company
Anti-RC3H1 antibody	ab70195	Abcam
Anti-β-actin antibody	P30002M	Abmart
Rabbit IgG	ab172730	Abcam
Pacific Blue™ anti-human CD45 Antibody	MHCD4528	eBioscienc
APC anti-human CD14 Antibody	367117	Biolegend
Pacific Blue™ anti-human CD45 Antibody	304022	Biolegend
FITC anti-human CD11c Antibody	337214	Biolegend
APC anti-human CD14 Antibody	367118	Biolegend
Anti-CK7 antibody	ZM-0071	ZSGB-BIO
Anti-CD14 antibody	ab181470	Abcam
Anti-CD31 antibody	ab28364	Abcam
HRP-secondary antibodies	128-035-160	Jackson
FITC-secondary antibodies	ZF-0311	ZSGB-BIO
TRICT-secondary antibodies	ZF-0313	ZSGB-BIO
Cyanine Cy™5-secondary antibodies	128-175-229	Jackson
