# Supplementary material for: MNSFβ Regulates TNFα Production by Interacting with RC3H1 in Human Macrophages, and Dysfunction of MNSFβ in Decidual Macrophages Is Associated With Recurrent Pregnancy Loss
Source: Front Immunol. 2021 Sep 13;12:691908. doi: 10.3389/fimmu.2021.691908 (PMC8473736; doi:10.3389/fimmu.2021.691908)
Supplement: Supplementary Table S4 — Proliferation of T-HESCs by Cell Viability Analysis. [file Table_4.pdf]

TABLE S4 | Proliferation of T-HESCs by Cell Viability Analysis

	**0 hr**	**24 hr**	**48 hr**
**siNC**	0.50E+04	1.94E+05 ± 7.48E+04	1.43E+05 ± 1.21E+04
**siMNSFβ**	0.50E+04	1.42E+05 ± 4.09E+04	1.20E+05 ± 2.09E+04
P value	N/A	> 0.05	< 0.05

(siNC: T-HESCs transfected by NC siRNA; siMNSFβ: T-HESCs transfected by the MNSFβ specific siRNA)
